# Metagenomic Study Reveals Phage–Bacterial Interactome Dynamics in Gut and Oral Microbiota in Pancreatic Diseases

**DOI:** 10.3390/ijms252010988

**Published:** 2024-10-12

**Authors:** Laura Vilà-Quintana, Esther Fort, Laura Pardo, Maria T. Albiol-Quer, Maria Rosa Ortiz, Montserrat Capdevila, Anna Feliu, Anna Bahí, Marc Llirós, Adelaida García-Velasco, Mireia Morell Ginestà, Berta Laquente, Débora Pozas, Victor Moreno, Librado Jesús Garcia-Gil, Eric Jeffrey Duell, Ville Nikolai Pimenoff, Robert Carreras-Torres, Xavier Aldeguer

**Affiliations:** 1 Digestive Diseases and Microbiota Group, Girona Biomedical Research Institute (IDIBGI), 17190 Salt, Spain; 2 Department of Gastroenterology, Hospital Universitari de Girona Dr. Josep Trueta, 17007 Girona, Spain; 3 Hepato-Pancreato-Biliary Unit, Department of Surgery, Hospital Universitari de Girona Dr. Josep Trueta, 17007 Girona, Spain; 4 Department of Pathology, Hospital Universitari de Girona Dr. Josep Trueta, 17007 Girona, Spain; 5 Bioinformatics and Bioimaging (BI-SQUARED) Research Group, Biosciences Department, Faculty of Sciences, Technology and Engineering, Universitat de Vic, 08500 Vic, Spain; 6 Precision Oncology Group, Girona Biomedical Research Institute (IDIBGI), 17190 Salt, Spain; 7 Institut Català d’Oncologia (ICO), Hospital Universitari de Girona Dr. Josep Trueta, 17007 Girona, Spain; 8 Hereditary Cancer Program, Institut Català d’Oncologia (ICO), Institut d’Investigació Biomèdica de Bellvitge (IDIBELL), CIBERONC, 08908 Barcelona, Spain; 9 Medical Oncology Department, Institut Català d’Oncologia (ICO), Institut d’Investigació Biomèdica de Bellvitge (IDIBELL), 08908 Barcelona, Spain; 10 Institut Català d’Oncologia (ICO), Institut de Recerca Biomedica de Bellvitge (IDIBELL), 08908 Barcelona, Spain; 11 UBICS, University of Barcelona (UB), 08028 Barcelona, Spain; 12 Consortium for Biomedical Research in Epidemiology and Public Health (CIBERESP), 08036 Barcelona, Spain; 13 Cancer Epidemiology Research Program, Unit of Nutrition and Cancer, Institut Català d’Oncologia (ICO), Institut d’Investigació Biomèdica de Bellvitge (IDIBELL), 08908 Barcelona, Spain; 14 Department of Clinical Science, Intervention and Technology—CLINTEC, Karolinska Institutet, 14152 Stockholm, Sweden; 15 Unit of Population Health, Faculty of Medicine, University of Oulu, 90220 Oulu, Finland

**Keywords:** metagenome analysis, gut microbiome, oral microbiome, virus, bacteria, phage–bacterial interactome, pancreatic diseases, chronic pancreatitis, pancreatic cancer

## Abstract

Individuals with pancreatic-related health conditions usually show lower diversity and different composition of bacterial and viral species between the gut and oral microbiomes compared to healthy individuals. We performed a thorough microbiome analysis, using deep shotgun sequencing of stool and saliva samples obtained from patients with chronic pancreatitis (CP), pancreatic ductal adenocarcinoma (PDAC), and healthy controls (HCs).We observed similar microbiota composition at the species level in both the gut and oral samples in PDAC patients compared to HCs, among which the most distinctive finding was that the abundance of oral-originated *Fusobacterium nucleatum* species did not differ between the oral and the gut samples. Moreover, comparing PDAC patients with HCs, *Klebsiella oxytoca* was significantly more abundant in the stool samples of PDAC patients, while *Streptococcus* spp. showed higher abundance in both the oral and stool samples of PDAC patients. Finally, the most important finding was the distinctive gut phage–bacterial interactome pattern among PDAC patients. CrAssphages, particularly *Blohavirus*, showed mutual exclusion with *K. oxytoca* species, while *Burzaovirus* showed co-occurrence with *Enterobacteriaceae* spp., which have been shown to be capable of inducing DNA damage in human pancreatic cells ex vivo. The interactome findings warrant further mechanistic studies, as our findings may provide new insights into developing microbiota-based diagnostic and therapeutic methods for pancreatic diseases.

## 1. Introduction

Pancreatic ductal adenocarcinoma (PDAC) is usually asymptomatic in the early stages and is often diagnosed (80–90% of cases) at an incurable advanced metastatic stage [1,2]. The small percentage of patients eligible for surgical resection and subsequent treatment often experience early recurrence. The mean age-standardized 5-year relative survival rate for PDAC among Europeans was estimated to be 6.9% in 2014 (EUROCARE-5 study), while a more recent estimation in 2020, only among people in Girona (Catalonia, Spain), was 7.05% [3,4]. In addition, chronic pancreatitis (CP), which is a condition characterized by longstanding inflammation of the organ with calcifications and exocrine insufficiency, has increased in incidence [5,6]. PDAC and CP are distinct diseases, but CP is considered a potential risk factor for PDAC [6,7]. The mechanisms underlying these disease outcomes are, however, not fully understood. Identifying these mechanisms is crucial for enhancing prevention strategies and developing targeted treatments to reduce the global burden of these diseases [2].

Over the past two decades, the advent of low-cost high-throughput parallel sequencing has allowed the contribution of the human microbiome to systemic diseases to be assessed [2,8,9,10]. Recent studies have revealed associations between gastrointestinal tract (GIT) neoplasms and specific gut microbiota profiles [11,12], leading to a better understanding of the role of gut microbes in cancer development [8,13]. However, the impact of the changes in the gut microbiome on PDAC tumorigenesis, progression, and drug resistance is still not clear [8]. Recent studies have highlighted the link between the human oral microbiome and pancreatic cancer development [2,14]. Studies have recently supported the association between poor oral health and the development of PDAC [15,16,17].

Most of these microbiome studies use the phylogenetically conserved 16S rRNA gene in prokaryotes for profiling microbial communities, which is methodically straightforward and cost effective. However, 16S rRNA gene classification is limited to bacterial genus and species-level profiles and might exclude all other non-bacterial taxa, such as Archaea, eukaryotes, and viruses [18]. Therefore, viruses can mainly be recovered through shotgun metagenomic sequencing [19]. Viruses, however, are often present and, thus, may play a relevant role in many diseases, although it is unknown to what extent [20]. The most abundant viruses among human microbes are bacteriophages, also known as bacterial viruses, and they account for >90% of the whole human virome [21]. Bacteriophages can be either temperate or lytic. Temperate phages play an important role in horizontal gene transfer, providing bacteria with new features that can be virulent to the human host. In contrast, strictly lytic phages have a central role in terms of the presence and abundance of specific bacterial taxa, through their target predating activity [22]. That is, phages may reveal important interaction patterns in order to explain bacterial dysbiosis related to GIT diseases [22]. A recent study, including different continental patient groups, revealed lower diversity in the gut and oral microbiota in PDAC patients than in healthy individuals, and identified a set of 30 gut and 18 oral species associated with PDAC in a Japanese cohort. Using these bacterial taxa, it was possible to predict the PDAC status of population cohorts from Spain and Germany. An important aspect to note was also the identification of phages that likely infected microbial species enriched in patients with PDAC, among the three cohorts [23]. Indeed, understanding the viral–bacterial interactome dynamics in the gut and oral microbiota of patients with pancreatic diseases can enhance the reproducibility of microbial signatures across different cohorts and inform new strategies to modulate the microbial composition linked to these diseases. In the present study, we conducted a thorough viral and bacterial microbiome analysis, using deep shotgun sequencing of stool and saliva samples obtained from patients with CP, PDAC, and healthy controls (HCs). Our objectives were to examine potential differences in the abundance of bacterial and viral taxa between the gut and oral microbiomes, as well as between individuals with different health statuses. Additionally, we aimed to explore interaction networks among the bacteria and bacteriophages within these groups. Our intention is to contribute to the development of microbiota-focused diagnostic tools and potential therapeutic approaches to pancreatic diseases.

## 2. Results

### 2.1. Microbial Diversity

**Figure 1:**
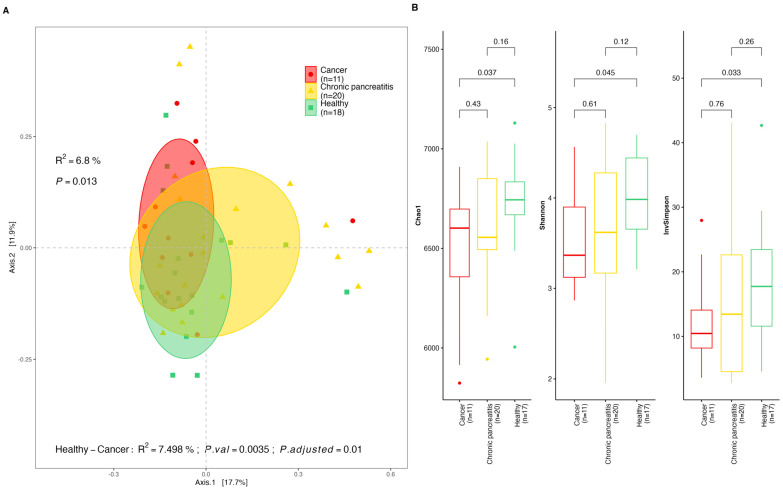
Diversity indexes for stool samples by diagnosis group. (**A**) Beta diversity principal coordinates analysis (*PCoA*) derived from Bray–Curtis distances among stool samples by group (cancer (PDAC), chronic pancreatitis (CP), and healthy controls (HCs)) with Bonferroni adjusted *p*-value for pairwise Adonis comparisons; (**B**) Alpha diversity indexes (Observed, Shannon, Inverse Simpson) for stool samples by study group (cancer (PDAC), chronic pancreatitis (CP), and healthy controls (HCs)).

**Figure 2:**
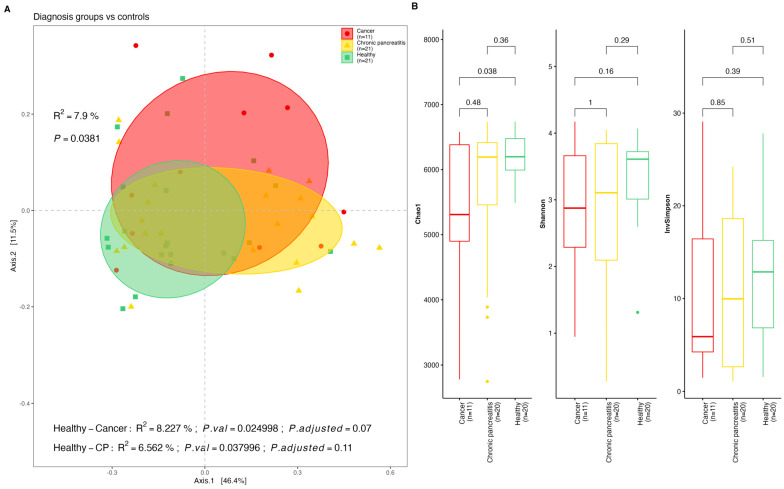
Diversity indexes for saliva samples by diagnosis group. (**A**) Beta diversity principal coordinates analysis (*PCoA*) derived from Bray–Curtis distances among saliva samples by group (cancer (PDAC), chronic pancreatitis (CP), and healthy controls (HCs)) with Bonferroni adjusted *p*-value for pairwise Adonis comparisons; (**B**) Alpha diversity indexes (Observed, Shannon, Inverse Simpson) for saliva samples by study group (cancer (PDAC), chronic pancreatitis (CP), and healthy controls (HCs)).

We observed differences in the microbial taxa distribution in stool and saliva samples between the CP and PDAC patients and the healthy controls. Beta diversity differences in terms of the microbial species distribution between the HCs and PDAC patients were observed (*p*-value = 0.013 for stools, Figure 1A; *p*-value = 0.038 for saliva, Figure 2A). This difference was sustained after the Bonferroni adjusted pairwise Adonis test was conducted for the stool samples (*p*-value = 0.01), but for the saliva samples, the difference was not significant after FDR correction (*p*-value = 0.07). The Alpha diversity indices within the groups revealed significantly higher microbial diversity in the stool samples among the HCs compared to the PDAC patients (Chao1, *p*-value = 0.037, Shannon, *p*-value = 0.045, InvSimpson, *p*-value = 0.033). However, there were non-significant differences between the HCs and the CP patients, and the PDAC patients and the CP patients (Figure 1B). The saliva samples from the same individuals showed a similar trend, but were non-significant in terms of the Shannon and InvSimpson indices (Figure 2B). Furthermore, we found the overall db-RDA test was significant for the diagnosis group variable to separate the CP patients and the PDAC patients from the healthy controls in terms of both the stool and saliva samples (*p* = 0.021 and 41.6% of the variance; *p* = 0.017 and 44.7% of the variance, respectively) (Figure S1).

### 2.2. Differential Abundance Analysis of Stool and Saliva Samples

Analysis of the representation of the whole gut and oral microbial community between the study groups was performed. Eighty-eight per cent (88%) of the taxa was shared between all the study groups, while 2.9% was only found in HC subjects, 2.7% in the CP patients, and 1.2% in the PDAC subjects (Figure S2). From the taxa, those with a relative abundance of less than 0.1% in the stool samples and 0.05% in the saliva samples, in 80% of all of the samples, were removed. A total of 84 species were found in the healthy controls to show differences in the relative abundance between the oral and the gut microbiota (Table S1). Slightly less in terms of the number of species (69) were identified for the CP patients (Table S2). However, only 49 species differed in abundance between the saliva and stool samples from the PDAC patients (Table S3). This decrease, together with the scattered distribution of the samples in Figure S3, may suggest a general shift in the gut microbial composition in pancreatic patients, especially for PDAC patients, towards oral microbiota composition.

Most importantly, we identified significant differences in the relative abundance at the genus level in the gut and oral microbiota composition between healthy individuals and PDAC patients. Mainly, *Veillonella* spp., *Prevotella* spp., *Porphyromonas* sp., *Neisseria* spp., *Haemophilus* sp., *Fusobacterium* spp., and *Parvimonas* sp., were more abundant in the oral cavity compared to the gut in the HCs (Table S1). Still, either a decreased number of species for each genus, or no differences between the oral cavity and gut, were seen in the PDAC patients (Table S3). Similarly, *Coprococcus* spp., *Blautia* sp., and *Alistipes* spp., were more abundant in the gut compared to the oral cavity in the HCs, but a decreased number of species, or no significant differences in the relative abundance, were seen for these genera between the oral cavity and the gut of PDAC patients (Tables S1 and S3).

### 2.3. Differential Abundance Analysis of the Groups of Patients

**Figure 3:**
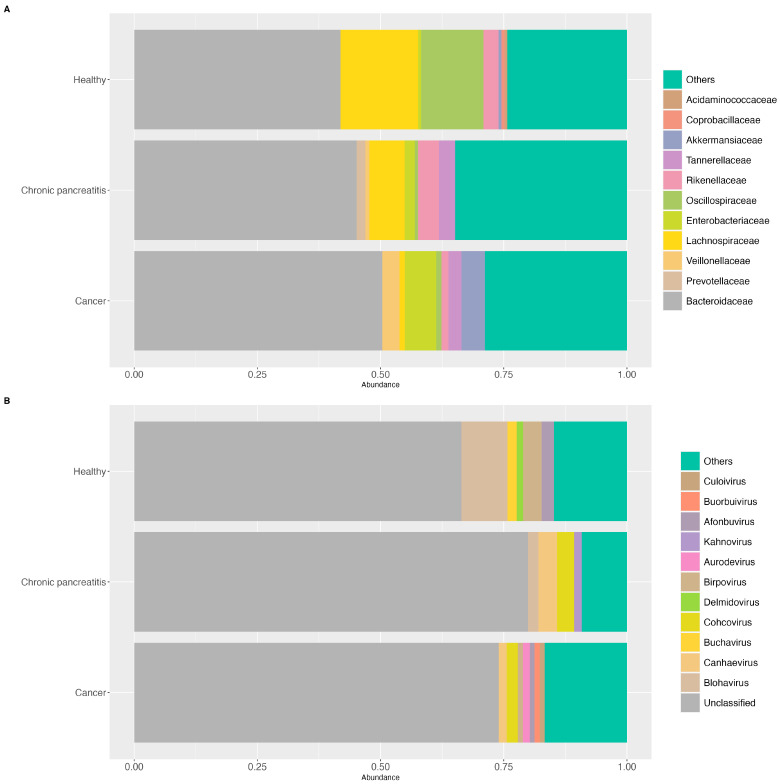
A graph of the 20% most prevalent bacterial and viral taxa in the stool samples among the groups. (**A**) A graph of the 20% most prevalent bacterial taxa in the stool samples agglomerated at the family level, according to the diagnosis group (cancer (PDAC), chronic pancreatitis (CP), and healthy controls (HCs)); (**B**) a graph of the 20% most prevalent viral taxa in the stool samples agglomerated at the genus level, according to the diagnosis group (cancer (PDAC), chronic pancreatitis (CP), and healthy controls (HCs)).

**Figure 4:**
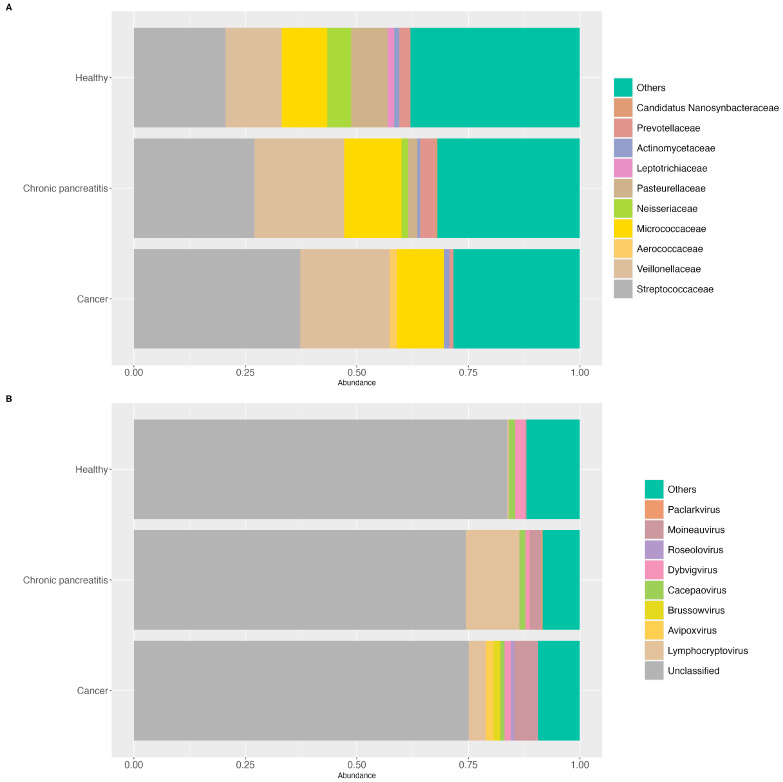
A graph of the 20% most prevalent bacterial and viral taxa in the saliva samples among the groups. (**A**) A graph of the 20% most prevalent bacterial taxa in the saliva samples agglomerated at the family level, according to the diagnosis group (cancer (PDAC), chronic pancreatitis (CP), and healthy controls (HCs)); (**B**) a graph of the 20% most prevalent viral taxa in the saliva samples agglomerated at the genus level, according to the diagnosis group (cancer (PDAC), chronic pancreatitis (CP), and healthy controls (HCs)).

For the stool samples, the most prevalent bacterial families in PDAC patients were *Bacteroidaceae*, *Veillonellaceae*, *Enterobacteriaceae,* and *Akkermansiaceae*, while the presence of *Culoivirus*, *Buorbuivirus*, *Aurodevirus*, among other viral genus, was a characteristic of this group. In the CP patients, the most prevalent bacterial families were *Bacteroidaceae*, *Prevotellaceae*, *Rikenellaceae,* and *Tannerellaceae*, and the viral genera *Canhaevirus* and *Cohcovirus*. In the HCs, the most prevalent bacterial families were *Bacteroidaceae*, *Lachnospiraceae,* and *Oscillospiraceae,* and the viral genus *Birpovirus* and *Blohavirus* (Figure 3A and Figure 3B, respectively). Regarding the saliva samples, *Streptococcaceae* and *Veillonellaceae* were the most prevalent bacterial families in all three groups. Nonetheless in the HCs, *Neisseriaceae* and *Pasteurellaceae* were more abundant. A higher abundance of *Micrococcaceae* was found in both PDAC and CP patients, while a higher abundance of *Prevotellaceae* was found in CP patients. On the other hand, regarding the viral genus, *Moineauvirus* and *Lymphocryptovirus* were amongst the most prevalent viral genus in the PDAC and CP patients, respectively (Figure 4A and Figure 4B; respectively). The differential abundance analysis of ALDEx2, adjusted for sex and age, revealed three bacterial species from the *Lachnospiraceae* family were significantly depleted (effect size = −1.38, −1.53, −1.30) and two bacterial species from the *Enterobacteriaceae* (effect size = 0.78) and *Streptococcaceae* families (effect size = 1.13) were enriched in the fecal samples of PDAC patients compared to the HCs. Also, one bacterial species from the *Faecalibacterium* genera (effect size = −0.71) was significantly more abundant in the HCs compared to the CP patients. Regarding the oral samples, three bacterial species from the *Streptococcaceae* family (effect size 0.64, 0.69, 0.81) were significantly more abundant, and one species from the *Pasteurellaceae* family (effect size −1.14) was depleted, in the PDAC patients compared to the HCs. Additionally, one bacterial species showed higher abundance in the CP patients compared to the HCs (effect size 1.07) (Table 1). Nonetheless, when adjusted for all the covariates (i.e., sex, age, tobacco smoking, and alcohol consumption), only the depletion of *Faecalibacterium* spp. in the CP patients remained significant in the stool samples. For the oral samples, only the enrichment of *Streptococcus* sp. *FDAARGOS_192* and *Streptococcus* sp. *HSISS3* in the PDAC patients remained significant.

### 2.4. Gut Microbe Interaction Analyses

**Figure 5:**
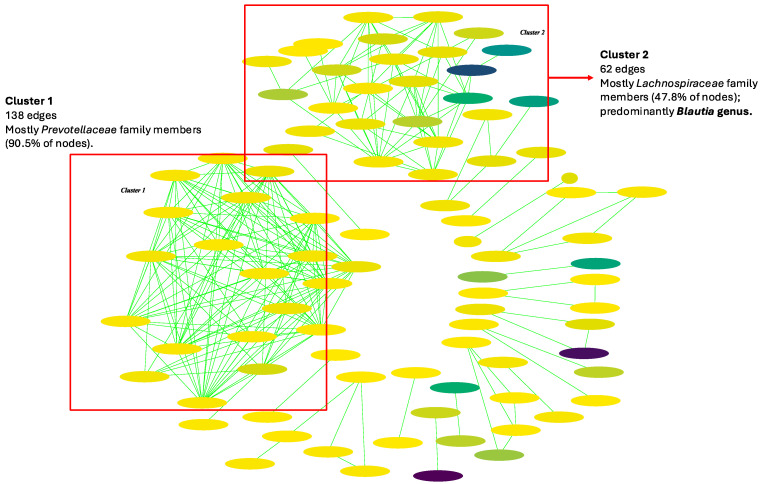
Gut microbial interaction network in the healthy controls (HCs). Green interactions represent co-occurrence relationships. The color gradient represents the relative abundance of the species in the samples (from low to high abundance) and the width represents the prevalence.

**Figure 6:**
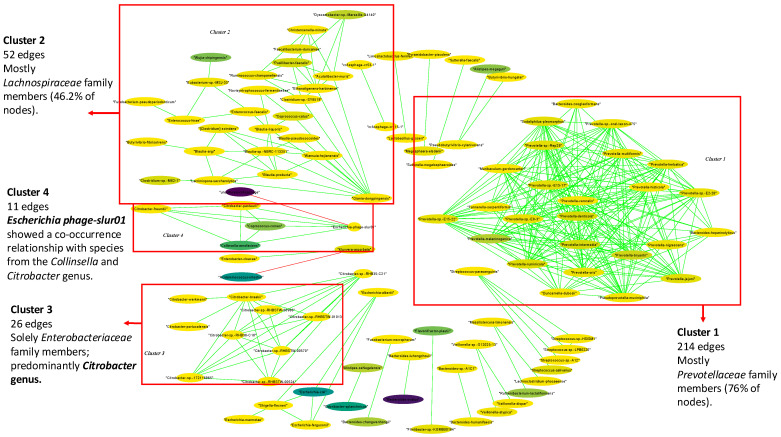
Gut microbial interaction network in chronic pancreatitis (CP) patients. Green interactions represent co-occurrence relationships, while red interactions represent mutual exclusion. The color gradient represents the relative abundance of the species in the samples (from low to high abundance) and the width represents the prevalence.

Interaction analyses were performed for each health status group in terms of the subjects. We observed the same two main clusters in the interaction analyses for the stool samples from both the HCs and CP patients (Figure 5 and Figure 6, Tables S4 and S6). These two clusters were comprised only of co-occurrence relationships, which we can interpret as two mutual clusters. The biggest cluster (Cluster 1) (138 and 214 edges for HCs and CP patients, respectively) was mostly comprised of species from the *Prevotellaceae* family (90.5% and 76% of the nodes, respectively). The second cluster (Cl. 2) (62 and 52 edges, respectively) was mostly comprised of species from the *Lachnospiraceae* family (47.8% and 46.15% of the nodes, respectively). The main difference was in the second cluster, where for the HCs, the *Blautia* genus was the most predominant among the *Lachnospiraceae* family, while in the CP network, the genera *Coprococcus*, *Wujia*, *Butyrivibrio*, and *Novisyntrophococcus* were the most abundant. In the CP network, a cluster with 26 edges was present (Cl. 3). This cluster was formed solely of species from the *Enterobacteriaceae* family, particularly the *Citrobacter* genus (100% of the nodes). Another small cluster was formed (11 edges) (Cl. 4), where *Escherichia phage-slur01* showed a negative correlation with the *uncultured CrAssphage* (*p*-value < 0.05), indicating the mutual exclusion of these two taxa (Table S7). At the same time, in this cluster, *Escherichia phage-slur01* showed a co-occurrence relationship with species from the *Collinsella* and *Citrobacter* genus (*p*-value < 0.05 in all cases) (Table S7). The observed differences in the interaction clusters in the HC and CP networks were present in smaller clusters (<=5 edges). Among them, we observed the same connection involving virus taxa, like the positive interaction between *CrAssphage cr115-1* and *CrAssphage cr53-1* (*p*-value < 0.05), indicating a pattern of co-occurrence between the taxa (Tables S5 and S7). *CrAssphage cr-53-1* was the most abundant viral species (0.13% of relative abundance) among the HCs.

**Figure 7:**
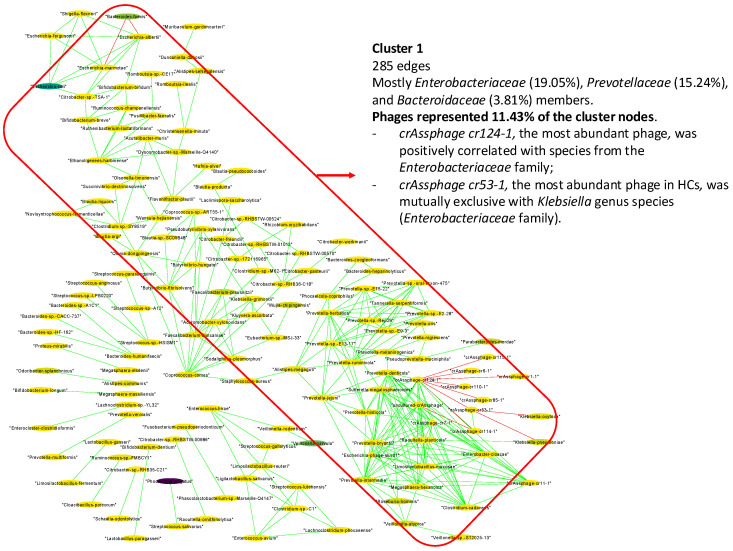
Gut microbial interaction network in pancreatic cancer (PDAC) patients. Green interactions represent co-occurrence relationships, while red interactions represent mutual exclusion. The color gradient represents the relative abundance of the species in the samples (from low to high abundance) and the width represents the prevalence.

Regarding the interaction analysis of PDAC stool samples, we observed a strikingly different pattern compared to the other two stool sample groups (Figure 7). The results showed a single predominant cluster (Cl. 1) (285 edges) that mostly integrated species from the *Enterobacteriaceae* (19.05% of the nodes), *Prevotellaceae* (15.24%), and *Bacteroidaceae* (3.81%) families (Table S8). *Escherichia coli* and *Bacteroides faecis* were the most abundant species present in the cluster (5.44%, 2.49% of the relative abundance, respectively). Interestingly, a mutual exclusion relationship was observed between the *Bacteroides faecis* and *Escherichia* genus species (*p*-value < 0.05 in all cases) (Table S9). In contrast to the other groups, the phages had a significant role in the interaction network in the PDAC samples. Phages represented 11.43% of the cluster nodes, including *crAssphage cr124-1*, the most abundant viral species (0.07% relative abundance) (Table S8). This phage showed a mutual exclusion relationship with *crAssphage cr53-1* (*p*-value < 0.05), the most abundant phage in HC samples (Table S9). Moreover, *crAssphage cr53-1* was mutually exclusive with the *Klebsiella* genus species (*Enterobacteriaceae* family) (*p*-value < 0.05 in all cases), which was only found in the PDAC network. The most abundant phage, *crAssphage cr124-1*, showed mutual exclusion with *CrAssphages cr1-1*, *cr114-1*, *cr85-1*, *cr110-1,* and cr*6-1*, but co-occurrence occurred with *CrAssphage cr11-1* and *crAssphage 7-1* (*p*-value < 0.05 in all cases). Finally, *crAssphage cr124-1* and the co-occurrence phages were positively correlated with species from the *Enterobacteriaceae* family, *Raoultella planticola*, and *Enterobacter cloacae* (*p*-value < 0.05) (Table S9).

### 2.5. Oral Microbe Interaction Analyses

**Figure 8:**
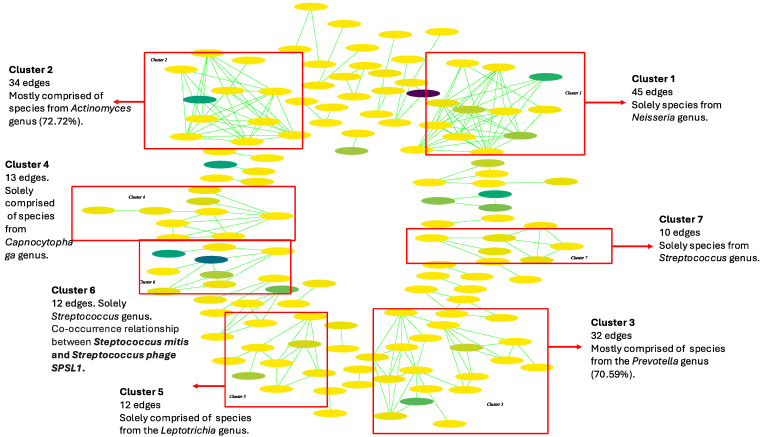
Oral microbial interaction network in the healthy controls (HCs). Green interactions represent co-occurrence relationships. The color gradient represents the relative abundance of the species in the samples (from low to high abundance) and the width represents the prevalence.

**Figure 9:**
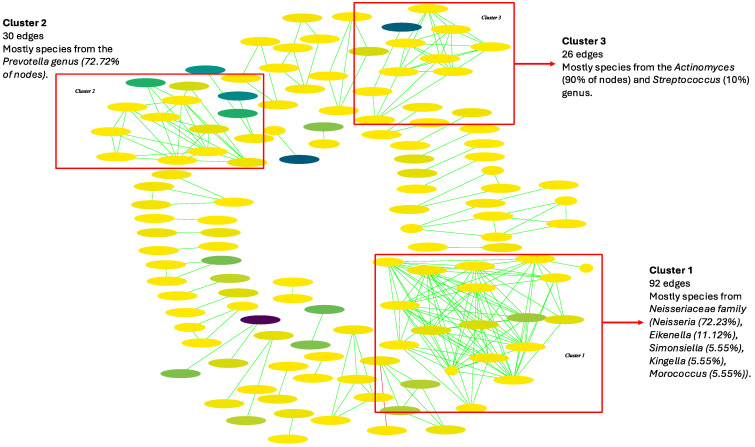
Oral microbial interaction network in chronic pancreatitis (CP) patients. Green interactions represent co-occurrence relationships, while red interactions represent mutual exclusion. The color gradient represents the relative abundance of the species in the samples (from low to high abundance) and the width represents the prevalence.

Three big microbial clusters of interaction were observed among the saliva samples from the HC group (Figure 8). The most diverse cluster (Cl. 1) (45 edges) comprised only of species from the *Neisseria* genus. The second most diverse cluster (Cl. 2) (34 edges) was mainly formed of species from the *Actinomyces* genus (72.72% of nodes), and the third cluster with 32 edges (Cl. 3) was mostly comprised of species from the *Prevotella* genus (70.59%). Smaller clusters with 13 and 12 edges (Cl. 4, Cl. 5), only involving species from the *Capnocytophaga* and *Leptotrichia* genera, respectively, were observed. Finally, *Streptococcaceae* family members were distributed in two clusters with 12 and 10 edges, respectively (Cl. 6, Cl. 7) (S10 Table). In the first one, we observed the only viral taxa interaction in the whole network, which was a co-occurrence relationship between *Streptococcus mitis* and *Streptococcus phage SPSL1* (*p*-value = 0.0018) (Table S11). The rest of the observed interactions were represented in minor clusters (<=6 edges). The three main clusters observed in the HC network were also present in the interaction analysis of the CP samples, although slight differences were observed (Figure 9, Table S12). The most significant cluster (92 edges) was solely comprised of species from the *Neisseriaceae* family (Cl. 1). However, more genera were represented in addition to *Neisseria* (72.23% of the nodes), such as *Eikenella* (11.12%), *Simonsiella* (5.55%), *Kingella* (5.55%), and *Morococcus* (5.55%). The second biggest cluster, in this case, had 30 edges and was mainly formed of species from the *Prevotella* genus (72.72% of the nodes) (Cl. 2). In comparison, the third cluster (26 edges) was comprised of species from the *Actinomyces* (90% of the nodes) and *Streptococcus* (10%) genus (Cl. 3). The rest of the observed interactions were clustered in minor groups with </= 10 edges. Interestingly, no interactions involving viral taxa were found in the CP network (Table S13).

**Figure 10:**
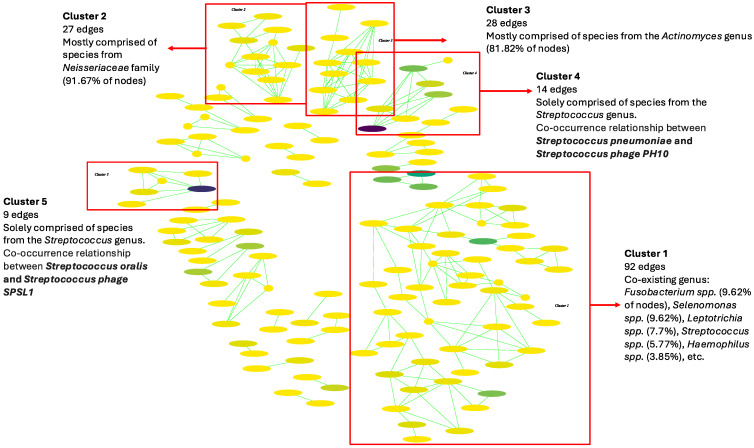
Oral microbial interaction network in pancreatic cancer (PDAC) patients. Green interactions represent co-occurrence relationships. The color gradient represents the relative abundance of the species in the samples (from low to high abundance) and the width represents the prevalence.

A major change in the pattern in the interaction network of the PDAC group was observed (Figure 10). One big cluster (Cl. 1) with 92 edges was observed as a combination of different coexisting genera, such as *Fusobacterium* spp. (9.62% of the nodes), *Selenomonas* spp. (9.62%), *Leptotrichia* spp. (7.7%), *Streptococcus* spp. (5.77%), and *Haemophilus* spp. (3.85%), among others (S14 Table). Moreover, genera, such as *Campylobacter*, *Anaerococcus*, and *Parvimonas,* were present in this cluster, while they were not represented in the HC network. An interesting interaction in terms of this cluster was the co-occurrence relationship between *Fusobacterium nucleatum* and *Campylobacter showae* (*p*-value = 1.01 × 10^−4^) (Table S15). In addition, a cluster with 27 edges, mostly comprised of species from the *Neisseriaceae* family (91.67% of the nodes) (Cl. 2), and a cluster with 28 edges, mainly consisting of species from the *Actinomyces* genus (81.82%) (Cl. 3), was observed, as in the other groups. However, the cluster made predominantly of the *Prevotella* genus was no longer present (Figure 10, Table S14). *Streptococcaceae* family members were observed in different minor clusters across the network (Table S14). In one of these clusters (13 edges) (Cl. 4), a co-occurrence relationship between *Streptococcus pneumoniae* and *Streptococcus phage PH10* was observed (*p*-value = 0.0018). Similarly, in another *Streptococcaceae* cluster with nine edges (Cl. 5), *Streptococcus oralis* showed a coexistence relationship with *Streptococcus phage SPSL1* (*p*-value = 0.0016) (Table S15). The rest of the interactions between the taxa were distributed in small groups with </= 6 edges.

### 2.6. Sensitivity Analysis

The diversity analysis conducted using the sensitivity database revealed a similar overall pattern. Moreover, similar to the initial results, common viral species were observed in the oral cavity, such as *Streptococcus phages*, and in the gut cavity, including *Blohavirus*, *Birpovirus*, *Culoivirus*, *Kahnovirus*, and *Delmidovirus*, among others.

Regarding the interaction analysis, the sensitivity analysis demonstrated a pattern similar to the one originally described (Table S16). Notably, we found that the *Blohavirus* genus is present in the healthy network and mutually exclusive with *Klebsiella* spp. in the cancer network. Additionally, the *Burzaovirus* genus is prevalent in the cancer network and shows positive interactions with species that promote inflammation.

In our sensitivity analysis, we utilized a newer, more extensively curated, microbial reference database (more refined and a larger number of reference genomes), which resulted in updates to the nomenclature of several viral species. These changes have been duly noted and are reflected in our analysis as follows: CrAssphage cr53-1 as *Blohavirus americanus*, CrAssphage cr110-1 as *Delmidovirus intestinihominis*, CrAssphage cr7-1 as *Burzaovirus coli*, CrAssphage cr124-1 as *Burzaovirus faecalis*, CrAssphage cr11-1 as *Delmidovirus splanchnicus*, CrAssphage cr85-1 as *Kahnovirus oralis*, CrAssphage cr115-1 as *Birpovirus hibernieae*, CrAssphage cr114-1 as *Aurodevirus intestinalis*, Escherichia phage slur 01 as *Unclassified Seuratvirus*, Streptococcus phage PH10 as *Streptococcus oralis phage PH10,* and Streptococcus phage SpSL1 as *Streptococcus phage SpSL1*. Considering this, *Blohavirus* remains a prevalent genus among the significant interactions in healthy individuals, positively interacting with members of the *Lachnospiraceae* family. Similarly, the *Burzaovirus* genus is observed in various interactions within the cancer group, interacting with members of the *Veillonelaceae*, *Lactobacillaceae*, and *Prevotellaceae* families. In regard to the oral networks, significant associations in the cancer group predominantly involved the *Pandoravirus* species, a dsDNA virus identified in our study as one of the most prevalent among the cancer patients.

## 3. Discussion

In the present study, we performed comprehensive microbial diversity and taxonomic distribution analysis of deep-sequenced metagenomes from the saliva and stool samples of patients with pancreatic diseases (PDAC and CP) and healthy controls. We compared microbial diversity indices and abundances, and the interaction networks in terms of coexistence and mutual exclusion, of bacterial and virus taxa between the groups of cancer patients and the healthy controls.

The most important findings from our study were that both the saliva and stool microbiota from the PDAC patients were similar to each other and were less diverse than the observed microbiota among the healthy individuals. This may indicate that there is an oral–gut migration of certain species, due to the gut barrier dysfunction that may contribute further to the pathogenesis of PDAC, as is observed for *F. nucleatum*. This is in agreement with previous reports that revealed a more diverse and balanced gut microbiota among healthy individuals. In contrast, dysbiosis of the gut microbiota is consistently observed in people with different comorbidities [24]. Our interactome analysis also revealed strikingly significant differential interaction patterns between the bacteria and phages, particularly among the gut microbiota of PDAC patients compared to the gut of healthy individuals.

We observed that PDAC patients showed a more similar composition in their oral and gut microbiota than healthy individuals, and a reduction in the diversity or richness in the oral cavity in PDAC patients compared to healthy individuals. As Park et al. [25] described, the oral and the gut microbiome profiles are well segregated due to the oral–gut barrier, physical distance, and chemical hurdles, such as the presence of gastric acid and bile. The gut microbiota of healthy subjects commonly comprises five major phyla dominated by Firmicutes and Bacteroidetes, which account for more than 90% of the microbiota [26]. At the same time, commensals in the oral cavity contain *Firmicutes*, *Proteobacteria*, *Bacteroidetes*, *Actinobacteria*, *Fusobacteria*, *Neisseria*, and TM7 [25]. However, the oral microbiota can translocate to the intestinal mucosa, as well as inversely, in conditions of oral–gut barrier dysfunction, which can be caused by medication intake, such as proton pump inhibitors (PPIs), advanced age, or chronic inflammatory conditions due to systemic disease. Higher relative abundances of the oral taxa *Fusobacterium* and *Porphyromonas* have been described in both the pancreatic and intestinal microbiomes of PDAC patients [27]. Also, *Veillonella* spp. has been widely reported to be enriched in the gut microbiome of PDAC patients compared to healthy subjects [23,28]. Thus, certain oral microbes might migrate to the gut and promote PDAC pathogenesis, by coordinating intestinal and pancreatic microbiome modulation [25]. Nonetheless, although a high abundance of the oral-originated *Parvimonas micra* in the gut of colorectal cancer (CRC) patients has been associated with the disease, no conclusive results have been published regarding the role of this species in the pathogenesis of PDAC [25].

Regarding the gut microbiota composition, we can highlight that members of the Firmicutes phyla (*Coproccocus* spp., *Butyrivibrio* spp. *(Lachnospiraceae),* and *Faecalibacterium* spp. *(Oscillospiraceae))* were significantly more abundant in healthy subjects compared to the groups with pancreatic diseases (PDAC, CP). At the same time, *Enterobacteriaceae* (Proteobacteria phyla), *Bacteroidaceae* (Bacteroidetes phyla), and *Veillonellaceae* (Firmicutes phyla) families were among the most prevalent in PDAC subjects. *Klebsiella oxytoca (Enterobacteriaceae)* and *Streptococcus anginosus* were significantly more abundant when compared to the HCs. Along with our results, the relative abundance of Proteobacteria has been reported to be significantly higher in the feces of patients with PDAC compared to the controls [29]. As previously described, short-chain fatty acid (SCFA) producers, such as *Lachnospiraceae* and *Oscillospiraceae*, are significantly less abundant in the gut microbiota of PDAC patients. *Enterobacteriaceae* proliferation has been reported to be promoted by PPAR-y inactivation, which results from a lack of SCFA and, subsequently, the higher available oxygen requirement for microbiota at the proximal mucosa [27]. Similarly, in the multinational study by Nagata et al. [23], dysbiosis of the gut microbiome was reported, and significant associations were identified between PDAC and 30 gut bacterial species, in which both *Streptococcus* and *Veillonella* spp. were described. Interestingly and according to the present study, Nagata et al. [23] found *S. anginosus* to be consistently enriched in the guts of patients with PDAC in Spanish and German cohorts. Moreover, some species of the *Bacteroidaceae* family have been reported to assist *Escherichia coli* in improving tumorigenic effectiveness, via triggering damage to double-stranded DNA [27]. Nonetheless, controversial results are found in the literature regarding *Bacteroidaceae*, as they have been widely reported as mutualistic bacteria in the human gut, present at high densities, and that perform beneficial functions for the human host, such as immunomodulation, colonization resistance against invading pathogens, the biosynthesis of vitamins, and cooperation with other commensal and mutualistic microbes [30].

In the gut interaction analyses, we observed two main clusters of co-occurrence relationships in both the HC and CP stool samples, which can be interpreted as two mutual clusters, mostly comprised of species from the *Prevotellaceae* family (first cluster) and species from the *Lachnospiraceae* family (second cluster). In contrast, the gut interaction network in PDAC patients showed a strikingly different pattern, with a main cluster of other bacterial families, where *Bacteroides faecis* (*Bacteroidaceae* family) was found to be negatively correlated with *Escherichia* spp. (*Enterobacteriaceae* family), which we can interpret as a mutual exclusion relationship. *Bacteroides faecis* have been principally described as a commensal microbe isolated from the feces of healthy adults, while *Escherichia* spp. is a well-known colonizing genus that expresses amyloid fibers thought to mediate surface and cell–cell contacts that promote biofilm formation and, hence, host colonization [31,32]. Also, a co-occurrence was found for the *Burzaovirus* (*CrAssphage cr124-1*, *cr7-1*) and *Delmidovirus* (*CrAssphage cr11-1*) genus with *Enterobacteriaceae* family members, specifically *Enterobacter cloacae* and *Raoultella planticola*, with the latter being described as very similar to *Klebsiella* genus members [33]. CrAssphages are prevalent phage members, strictly lytic, within the Crassvirales order. Their known bacterial hosts have been widely reported as members of the phylum Bacteroidetes, mainly the *Bacteroidaceae* family [30]. Although crAssphages are hypothesized to be stable colonizers in the human gut, their linkage to human health and disease remains unclear [21]. It can be hypothesized that *Bacteroidaceae* family members are less abundant in this network cluster due to the presence of crAssphages and their host interaction with this family, which may facilitate the proliferation of *Enterobacteriaceae*. Nonetheless, a mutual exclusion relationship was found between *Klebsiella spp., specifically, K. oxytoca* and *K. pneumoniae*, and the *Blohavirus* genus (*CrAssphage cr53-1*). *Blohavirus* was the most prevalent viral genus found in healthy individuals, along with the *Birpovirus* genus (*CrAssphage cr115-1*), which were found to coexist in the network of healthy subjects and were mutually exclusive with *Burzaovirus* (*CrAssphage cr124-1*) in the PDAC network. A recent study cultivated the microbiome from pancreatic cyst fluid samples of intraductal papillary mucinous neoplasms (IPMNs), which are known precursors to PDAC, and found that *Gammaproteobacteria* (Proteobacteria phylum) members were dominate among the individual bacteria isolates [34]. Of these, several *Klebsiella* spp. and *Enterobacter cloacae* were repeatedly found and were shown to be capable of inducing DNA damage in human pancreatic cells ex vivo [34]. Other studies have described *Klebsiella* spp. as starvation tolerant in mucin-rich environments and, specifically, *K. oxytoca* has been linked to cancer, as it has been proven to increase in cancer cachexia cases [34,35,36]. A recent analysis of diverse, circular crass-like phage genomes has revealed some unusual aspects of their architecture and biology. Most crass-like sequences have been assigned to hosts in the phylum Bacteroidetes, although lower frequencies align with other bacterial phyla, such as gut Firmicutes and Proteobacteria [30,37]. Bacteriophage therapy has proven to be effective in combating bacterial biofilms and controlling bacterial infections, offering a complementary treatment to antibiotics. Phages can modulate the immune system during bacterial infections by enhancing phagocytosis, inducing cytokine responses in the innate immune system, and promoting antibody production in the adaptive immune system. They have also been shown to effectively treat antibiotic-resistant infections in immunocompromised cancer patients with solid tumors, while improving the immune response [38]. Bacteriophages have already been employed to treat pancreatitis caused by multidrug-resistant *Acinetobacter baumannii*, demonstrating their potential for addressing invasive bacterial infections in the pancreas [39]. While crass viruses likely play significant roles in shaping the human microbiome’s composition and functionality, the precise mechanisms involved remain largely unknown [40,41]. Advances in phage therapy have positioned it as an ideal compassionate treatment, especially given its lack of significant adverse effects.

Among the bacterial taxa showing a prominent role in the relationship between the oral microbiota and pancreatic disease, we observed the *Prevotellaceae* family related to CP patients and the *Streptococcus* genus associated with PDAC patients, as those families were among the most prevalent ones found in the oral cavities of the patients with such diseases, respectively. *Prevotellaceae* is recognized as one of the core anaerobic families in the oral microbiome. Nonetheless, members of the *Prevotella* genus belong to microbial communities in the gastrointestinal and respiratory tracts [42]. Studies indicate that *Prevotella* predominantly activates the Toll-like receptor 2, including interleukin-23 (IL-23) IL-1, and stimulates epithelial cells to produce IL-8, IL-6. Compared with strict gut commensal bacteria, *Prevotella* exhibits increased inflammatory properties, as demonstrated by the increased release of inflammatory mediators [25]. These findings align with our study on *Prevotella* in the CP group, as members of the genus may participate in human disease by promoting chronic inflammation [43].

Regarding the *Streptococcus* genus, previous studies have observed similar patterns than those observed in the current study [2,44,45]. However, contradictory results have been described for the oral performance of the *Streptococcus* genus, which did not allow us to reach a solid conclusion on the role of this taxon on PDAC [23,46]. In particular, in the oral interactome analyses, we found *S. mitis* and *S. oralis* coexisting with *Streptococcus phage SPSL1* in the HCs and PDAC patients, respectively. The *Streptococcus phage SPSL1* is an unclassified phage from the Caudoviricetes class. A recent study grouped *Streptococcus* spp. phages into a phylogenetic tree, in which *Streptococcus phage SPSL1* was grouped with phages found to be related mainly to *S. pneumoniae* [47]. Genetic similarities and the sharing of genes encoding virulence factors have been observed among streptococcal species (i.e., *S. mitis*, S. *oralis*, and *S. pneumoniae*). This phenomenon is thought to be due to the shared evolutionary origin, the homologous recombination, and the horizontal gene transfer mechanisms between those streptococcal species residing in the same ecological niche [48,49]. Similarly, in the PDAC oral network, a coexistence relationship was found between *S. pneumoniae* and *Streptococcus phage PH10*. This phage was found to be released from *S. oralis* strains isolated from human dental plaque. The putative endolysin from PH10 was purified and shown to have lytic activity with *S. oralis*, *S. pneumoniae*, and *S. mitis,* but not with other streptococcal species [50]. Both described phages are temperate, known as lysogenic phages, which can initiate either the lytic or lysogenic cycle after infection, depending on the surrounding environmental conditions. In the lysogenic cycle, temperate phages integrate their genomes into bacterial chromosomes as a form of prophages, which can replicate, along with the bacterial genome, which, in those cases, could partly explain the positive viral–bacterial interactions observed [51]. More genomic characterization of bacteriophages is urgently needed in the context of pancreatic diseases, as limited studies are currently available [40]. To date, no studies have specifically evaluated the use of bacteriophages for the treatment of pancreatic cancer or their potential in modulating the associated microbiome [41].

The present study suggests that phage therapy may be a promising approach to modulating the microbiome, by eliminating certain species. Thus, further studies on the genomic information of phages to treat or prevent PDAC are needed. However, this study has several limitations. The sample size of the study groups was relatively small, highlighting the need for a larger sample size to validate the robustness of the results. We performed a power analysis based on the R^2^ values obtained from the PERMANOVA results of the beta diversity analysis using the Bray–Curtis distance. For the stool samples, we found a power of 0.35, indicating a limited ability to detect the observed effect (R^2^ = 0.068 or 6.8%) in the beta diversity analysis. For the saliva samples, the power was 0.44 (R^2^ = 0.079 or 7.9%). Ideally, we aimed for a power of 0.8 or higher, suggesting that there is a 35% chance of detecting a significant difference in the stool samples and a 44% chance for the saliva samples, if one exists. Although the observed power is low, it can be attributed to the relatively small effect sizes. In microbiome studies, it is common for the variance explained by groupings to be modest, often around 5–10%, and such effect sizes can still be biologically meaningful, even with lower statistical power. Nonetheless, the minimum sample sizes needed to achieve 80% power for this study are 135 for the stool samples and 115 for the saliva samples.

Additionally, the non-standardized methodology for managing salivary samples may have caused contamination. The use of proton pump inhibitors (PPIs) in the PDAC group was not accounted for due to the small sample size, which could have introduced bias. Furthermore, research should investigate the causal relationship between potential microbial signatures and the development and progression of PDAC. Moreover, the bacterial–viral interactome described in PDAC patients should be monitored in cancer risk assessments.

## 4. Materials and Methods

### 4.1. Study Design

The study cohort comprised 53 participants; 11 individuals with PDAC, 21 individuals with CP, and 21 healthy subjects (HCs). Participants were recruited at the Hospital Universitari Dr. Josep Trueta (HUJT; Girona, Spain), between September 2020 and June 2022. Sixty-two per cent of all the participants were males. In addition, demographic information, clinical features, and medication intake were documented for all the participants in the study. Information about tobacco smoking (current smoker, former smoker, or never smoked) was based on their cigarette consumption at the time of enrolment, and alcohol consumption (high, moderate, and low) was based on the number of standard alcoholic drinks per week (≥7 per week; between 2 and 7 per week; <2 per week, respectively) (Table 2). Familial pancreatic history was reported in 18.2% of the PDAC patients and 9.5% of the CP patients. Most of the recruited CP patients were diagnosed due to toxic habits (80.9%), while only 19.1% were diagnosed due to obstruction or idiopathic causes.

### 4.2. Sample Collection

A total of 49 stool samples and 53 saliva samples were used in the study. The stool samples from 4 participants (1 CP and 3 HCs) were excluded due to poor storage conditions after collection. Fecal samples were collected by the participants in sterile feces containers. Participants were provided with clear instructions: samples deposited immediately before the doctor’s appointment were to be kept at room temperature, while those deposited more than 6 h prior were stored at 4 °C, until delivery. Once received at the hospital, all samples were promptly aliquoted in 8.0 mL sterile tubes, with unique identification numbers, and stored at −80 °C to ensure preservation, until DNA extraction. Saliva samples were collected by participants in 10.0 mL sterile conical tubes and delivered to the hospital facilities at room temperature </= 24 h after collection. Participants were told to spit into the tube and collect a minimum of 2.0 mL of saliva. Participants were advised not to eat, drink, smoke, or chew gum for at least 30 min before producing the saliva sample. Samples were immediately aliquoted in 1.5 mL Eppendorf tubes, with unique identification numbers, and stored in a −80 °C freezer, until DNA extraction.

### 4.3. Metagenomic Sequencing

The total genomic DNA was purified from 200 mg of fecal and 1.0 mL of saliva samples. DNA extractions were performed using a GenElute stool DNA isolation kit (catalogue number DNB200; Sigma-Aldrich^®^, St. Louis, MO, USA) for the stool samples and a QIAmp DNA microbiome kit (catalogue number 51704, QIAGEN^®^, Hilden, Germany) for the saliva samples, following the manufacturer’s instructions, and eluted in 100 µL of Elution Buffer. The total genomic DNA was quantified using a Nanodrop ND-2000 UV–Vis spectrophotometer (Nanodrop, DE) and Qubit^®^ (ThermoFisher Scientific^®^, Waltham, MA, USA) measurements. Shotgun metagenomic sequencing was performed for all the samples at an external facility (Finnish Functional Genomics Centre (FFGC), www.utu.fi/en, accessed on 31 May 2024). Briefly, the quality of the samples was ensured using an Agilent Bioanalyzer 2100 or an Advanced Analytical Fragment Analyzer (Agilent Technologies^®^, Santa Clara, CA, USA). The sample concentration was re-measured to ensure the DNA quantity. Sixty nanograms (60 ng) of DNA and six nanograms (6 ng) from nine low DNA input samples were used for the library preparation, using the Illumina DNA Prep Library Preparation Tagmentation kit (catalogue index ID 20027213; Illumina^®^, San Diego, CA, USA), according to the library preparation protocol (reference guide nº 1000000025416; Illumina^®^). The libraries were sequenced using the paired-end method (2 × 150 base pair) on the Novaseq 6000 S4 platform (Illumina^®^), with an estimated output of 8000–10,000 M reads/run.

### 4.4. Classification of Microbial Taxa

The original paired-end metagenome sequences, obtained as fastq files, were quality-filtered using Trimmomatic and BBmap, including the removal of duplicate reads. Only high-quality reads (Q20) were retained for the microbial analysis. The total number of reads per sample, after the removal of low-quality and clonal reads, as well as the percentage of human-classified reads per sample, are provided in Supplementary Table S17.

The stool samples had a lower fraction of human reads (0.08–3.77%), while the saliva samples showed a higher, but highly variable, proportion of human reads in terms of the metagenomes (around 20–30% for most samples, except for two outliers around 70–90%). Microbial taxa classification was performed using a K-mer-based classification algorithm and a K-mer matrix, from the fully curated bacterial, fungal, viral, archaeal, and protozoan taxa in the RefSeq reference genome database (v.2023). Each classified species was evaluated based on the evenness of the classified reads and their coverage across the reference genome, with an in-house threshold applied to exclude potential false-positive taxa.

Compositional analyses were conducted based on the observed read counts for each taxon, with various log transformations applied, as described in the relevant sections.

### 4.5. Statistical Analyses

#### 4.5.1. Diversity and Compositional Differences

To estimate the diversity differences between the microbes at the species level within and between the sample groups, we estimated the alpha diversity indices (Chao1, Shannon, and InvSimpson) and beta diversity matrices, respectively. The normalized sample size was determined with a repeated rarefaction, without replacement [52]. For the alpha diversity, normality was assessed through the Shapiro–Wilk test, and statistical differences between the diagnosis groups were analyzed using the one-way analysis of variance (ANOVA) or the Kruskal–Wallis test. Additionally, pairwise comparisons between the groups were performed. Bray–Curtis distances were computed and plotted for the beta diversity matrices, using principal coordinate analyses (PCoA). A non-parametric PERMANOVA test implemented in the Adonis function of the vegan package in R, using 10000 permutations, was performed to recognize the statistical differences between the diagnosis groups. Then, pairwise Adonis comparisons were conducted to identify the statistically significant differences between the groups. The Bonferroni adjusted *p*-value method was used for the pairwise Adonis comparisons.

The taxa were filtered at 0.1% of relative abundance for the stool samples and 0.05% for the saliva samples, to represent the viral community in the differential abundance analyses appropriately. The taxa were then agglomerated at the family and genus levels, including only those with 20% prevalence in the abundance plots. Differential abundance analyses, adjusted by sex and age, were performed using the generalized linear model (glm) module of the ALDEx2 package in R. The package identifies differentially abundant features by generating Monte Carlo (MC) instances of the log-ratio transformed data based on the provided count matrix, using the Dirichlet distribution and applying univariate statistical models to the MC instances [53]. Lastly, it calculates the expected adjusted false discovery rate (FDR) *p*-values across all MC instances [53]. Significant differences were considered when the FDR-corrected *p*-value was <0.05. In addition, Bray–Curtis distance-based redundancy analysis (db-RDA) was performed using the capscale function of the vegan package in R to assess whether the changes in gut and oral microbiota could be correlated with clinical (i.e., diagnosis group, BMI, alcohol and tobacco consumption) and demographics data (i.e., age and gender). A Venn diagram was used to visually represent the relationship between the whole gut and oral microbial communities between the study groups. A t-test pairwise comparison was also performed using the ALDEx2 package in R to compare the microbiota composition of the stool and the saliva samples within each of the study groups: healthy controls, CP, and PDAC. Non-metric multidimensional scaling (NMDS) was used to illustrate the distribution pattern of the microbial community (both gut and oral) and the study groups (healthy controls, CP, or PDAC).

#### 4.5.2. Interactome Analyses

Finally, analysis of the microbial association networks was conducted to study the interaction (co-occurrence/mutual exclusion relations) between the species in each of the diagnosis groups, using CoNet (2017) with Cytoscape (v.3.10.1). Four different methods were applied to the ensemble inference: Pearson, Spearman, Mutual Information, and Bray–Curtis dissimilarity with the 1000 top edges, both positive and negative, for each method. Permutations were then carried out to compute the *p*-values. “EdgeScores” was selected, with “shuffle_rows” as a resampling parameter, and “renormalize” was enabled. This last option alters the computation of permutation distributions for correlation measures by introducing a renormalization step that mitigates the compositionality bias [54]. Significance was estimated from method-and-edge specific permutation and bootstrap score distributions. In the bootstrap distribution step, all method-specific *p*-values for an edge were merged into one *p*-value using Brown’s method [55]. Benjamini–Hochberg multiple testing correction was enabled. For the final network, edges with original scores outside the 0.95 range of the bootstrap distribution were discarded.

### 4.6. Sensitivity Analysis

In order to confirm the robustness of our results, we performed a sensitivity analysis using a more extensive database for viral classification of the taxa and conducted the same interaction network analyses across all the study groups.

We obtained a comprehensive reference database using Kmer matching (kraken) including the NCBI Viral Genome resource (https://ncbiinsights.ncbi.nlm.nih.gov/2023/10/19/changes-virus-data-resources-ncbi/, accessed on 31 May 2024) and the Eukaryotic Pathogen Genome Database (http://www.eupathdb.org/, accessed on 31 May 2024). These databases add up for the most extensive viral and bacterial data available. We applied initial filters, using the depth of coverage (total read hits in the taxa > 100) and the breadth of coverage (unique read hits in the taxa > 100) as thresholds.

## 5. Conclusions

We conducted a thorough microbiome analysis to assess whether the observed gut or oral microbial diversity alterations between individuals with pancreatic disease and healthy controls could be attributed to microbial interactions, specifically between bacteria and phages.

A loss of microbial diversity was described in both the saliva and stool microbiota of PDAC patients compared to HCs. Similar gut and oral microbiota composition was described in PDAC patients, suggesting the oral–gut migration of certain species due to gut barrier dysfunction, which may contribute to the pathogenesis of PDAC, as is the case for *F. nucleatum*. The interactome analysis revealed additional insights into how crass phages, specifically *Blohavirus* and *Burzaovirus*, may modulate the prevalence of *Enterobacteriaceae*, particularly *Klebsiella oxytoca*, while *Streptococcus* phages may influence the abundance of *Streptococcaceae* family members.

This systematic interactome analysis warrants further research as it may contribute to understanding the complex relationships between viral–bacterial microbes and their potential role in pancreatic diseases, providing new insights into the development of microbiota-focused diagnostic tools and potential therapeutic approaches.

## Figures and Tables

**Table 1:** Results of the differential abundant analysis of ALDEx2, adjusted for sex and age, for HCs vs. PDAC/CP in stool and saliva samples.

Groups Compared	Species	Group *p*-Value	Group Adjusted *p*-Value	Effect	Overlap
*Stool PDAC* vs. *HCs*	*Coprococcus* sp. *ART55/1*	0.0001	0.03	−1.38	0.09
	*Butyrivibrio fibrisolvens*	0.0002	0.05	−1.53	0.13
	*Butyrivibrio hungatei*	0.0001	0.03	−1.30	0.04
	*Streptococcus anginosus*	0.0002	0.05	1.13	0.09
	*Klebsiella oxytoca*	0.0002	0.04	0.78	0.27
*Stool CP* vs. *HCs*	*Faecalibacterium duncaniae*	0.0002	0.05	−0.71	0.22
*Saliva PDAC* vs. *HCs*	*Streptococcus* sp. *FDAARGOS_192*	0.0001	0.02	0.64	0.14
	*Streptococcus* sp. *HSISS3*	0.0002	0.05	0.69	0.17
	*Streptococcus thermophilus*	0.0001	0.03	0.81	0.18
	*Actinobacillus porcitonsillarum*	8.13 × 10^−5^	0.02	−1.14	0.05
*Saliva CP* vs. *HCs*	*Limosilactobacillus fermentum*	0.0002	0.04	1.07	0.12

Group *p*-value: non-FDR corrected *p*-value for the generalized linear model (glm). Group adjusted *p*-value: FDR corrected (Holm method) *p*-value for the generalized linear model (glm) (</=0.05). Negative effect size values determine higher relative abundances in the basal (0) group (HC group), while positive effect size values determine higher abundances in the effect (1) group (PDAC/CP group).

**Table 2:** Baseline characteristics of enrolled patients by groups.

	PDAC	CP	HCs	*p*-Value
Sex, % (F/M)	63.6/36.3	14.3/85.7	47.6/52.4	0.0075 **
Age, yrs	68.81 ± 9.90	57.71 ± 7.06	61.05 ± 10.17	0.0068 **
Tobacco smoking, % (current/former/never)	18.2/45.4/36.4	47.6/33.3/19.1	9.6/33.3/57.1	0.03 *
Alcohol intake, % (high/moderate/low)	18.2/9.1/72.7	0.0/80.9/19.1	4.8/4.8/90.4	0.0005 **
BMI, kg/m^2^	26.93 ± 6.47	24.95 ± 5.16	26.16 ± 4.68	0.59
Diabetes, % (yes/no)	36.4/63.6	61.9/38.1	4.8/95.2	0.0005 **
Insulin treatment, % (yes/no)	100/0	69.2/30.8	100/0	0.61

HCs: healthy controls (n = 21), PDAC: pancreatic cancer (*n* = 11), CP: chronic pancreatitis (*n* = 21). F: females, M: males. Parametric variables are expressed as mean ± SD for numerical data and in % for categorical data. Pearson’s chi-squared test is used for categorical data and the ANOVA test is used for numerical data. ** *p* < 0.005, * *p* < 0.05.

## Data Availability

Raw data were generated at the *Finnish Functional Genomics Centre* (FFGC), Turku, Finland. Derived data supporting the findings in this study are available from the corresponding authors on request. All sequences generated in the present study are available and accessible through National Center for Biotechnology Information (NCBI) bioprojects number: PRJNA1083602 and PRJNA1083779.

## Supplementary Materials

The following supporting information can be downloaded at: https://www.mdpi.com/article/10.3390/ijms252010988/s1.
