# Supplementary figures and images for: Metagenomic Study Reveals Phage–Bacterial Interactome Dynamics in Gut and Oral Microbiota in Pancreatic Diseases
Source: Int J Mol Sci. 2024 Oct 12;25(20):10988. doi: 10.3390/ijms252010988 (PMC11507633; doi:10.3390/ijms252010988)

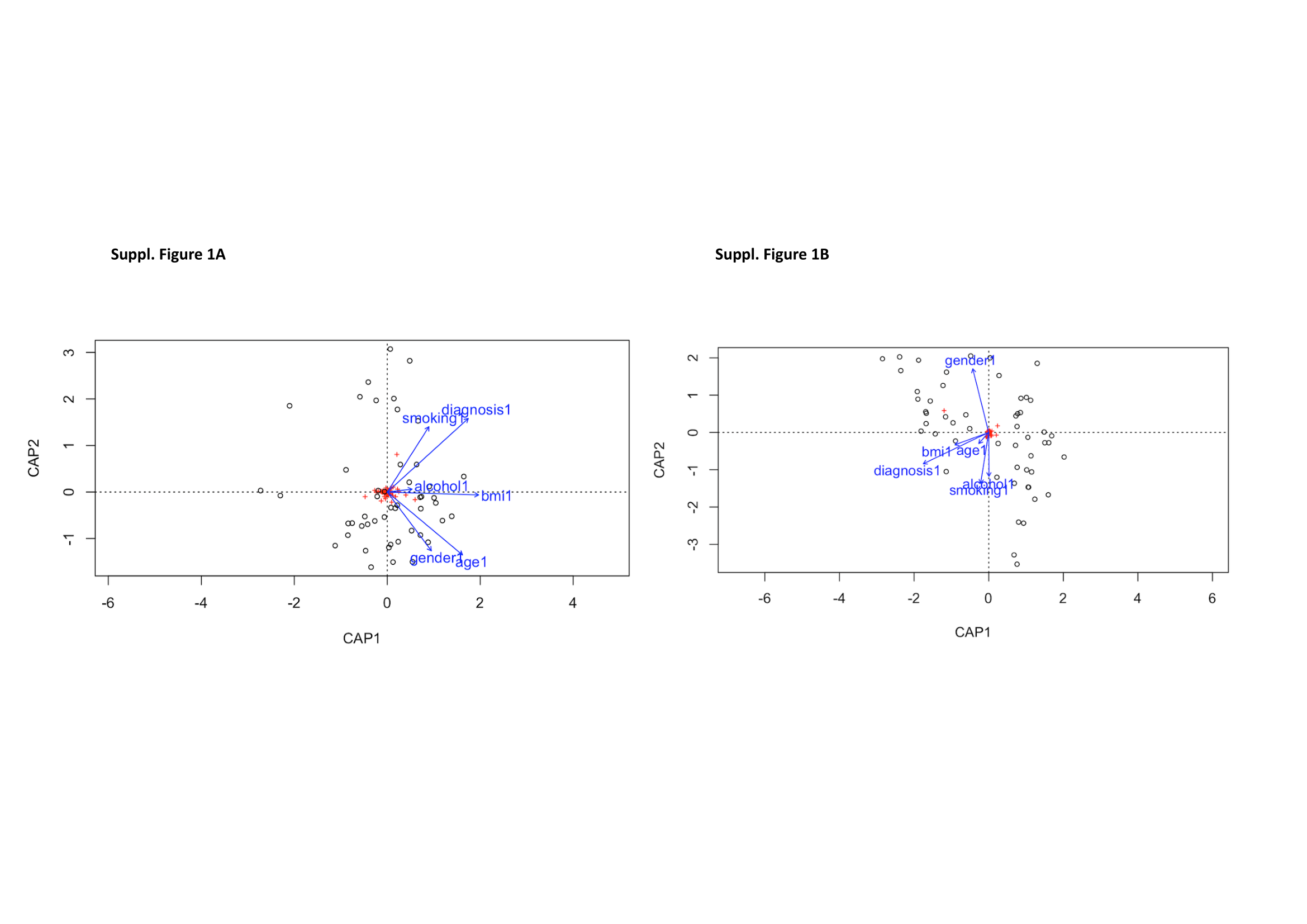

Supplement: Supplementary file 1 [file ijms-25-10988-s001.zip › S1Fig.tiff]

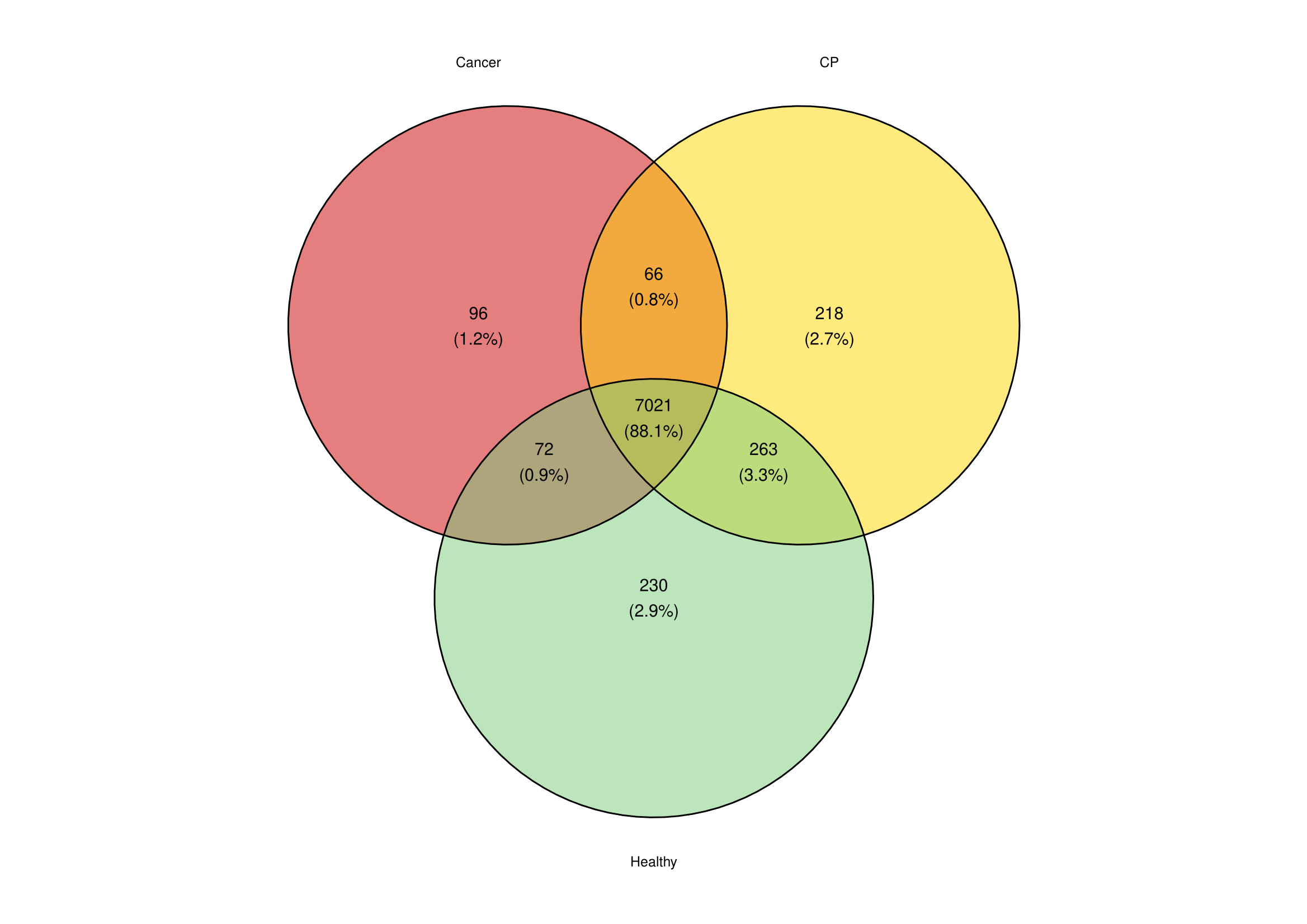

Supplement: Supplementary file 1 [file ijms-25-10988-s001.zip › S2Fig.tiff]

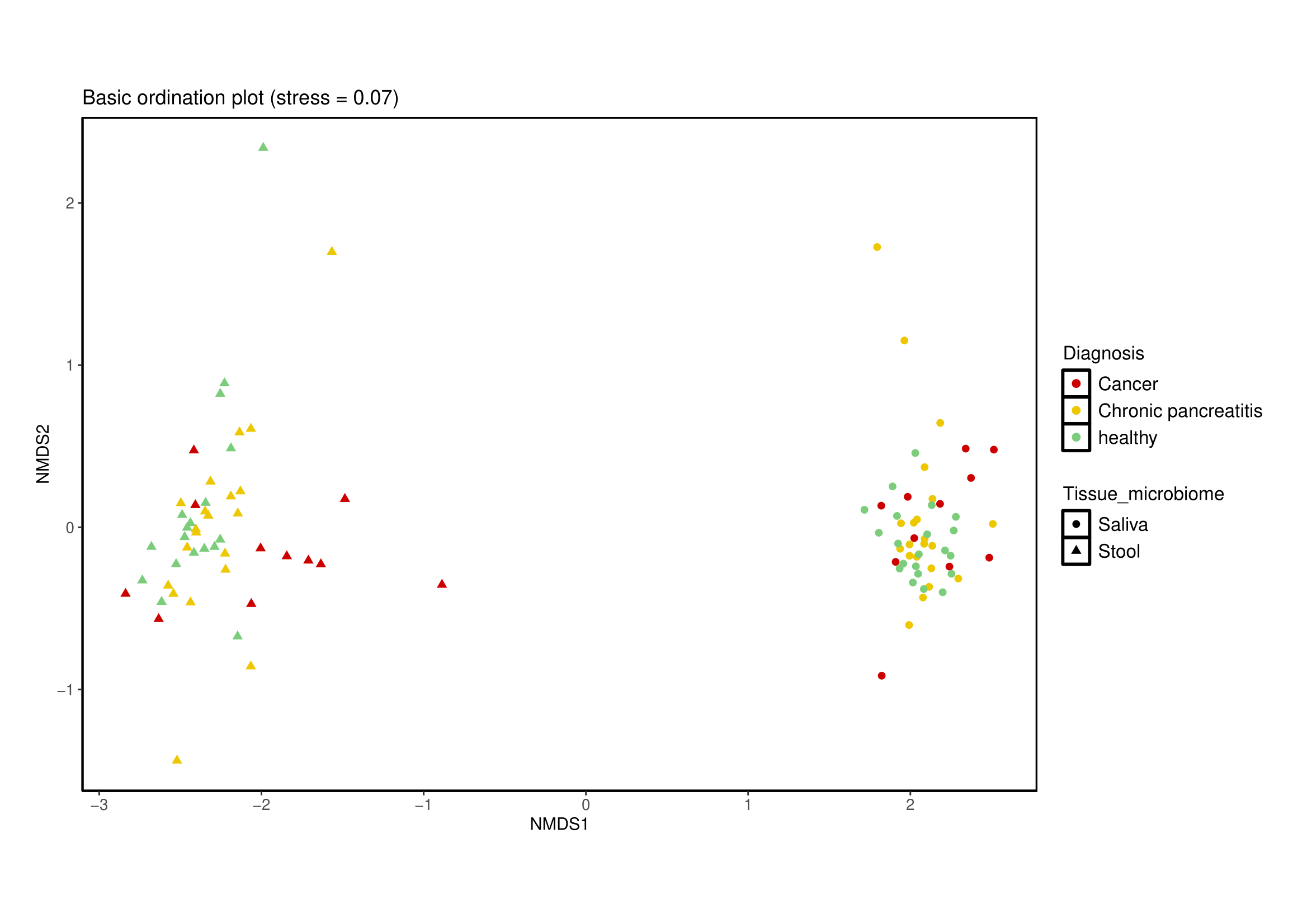

Supplement: Supplementary file 1 [file ijms-25-10988-s001.zip › S3Fig.tiff]
